# ProteinLIPs: a web server for identifying highly polar and poorly packed interfaces in proteins

**DOI:** 10.1093/bioinformatics/btaf499

**Published:** 2025-09-10

**Authors:** Helena García-Cebollada, Alfonso López, Vladimir E Angarica, Juan José Galano-Frutos, Javier Sancho

**Affiliations:** Biocomputation and Complex Systems Physics Institute (BIFI)-Joint Unit GBsC-CSIC, University of Zaragoza, 50018 Zaragoza, Spain; Departamento de Bioquímica y Biología Molecular y Celular, Facultad de Ciencias, University of Zaragoza, 50009 Zaragoza, Spain; Biocomputation and Complex Systems Physics Institute (BIFI)-Joint Unit GBsC-CSIC, University of Zaragoza, 50018 Zaragoza, Spain; Biocomputation and Complex Systems Physics Institute (BIFI)-Joint Unit GBsC-CSIC, University of Zaragoza, 50018 Zaragoza, Spain; Departamento de Bioquímica y Biología Molecular y Celular, Facultad de Ciencias, University of Zaragoza, 50009 Zaragoza, Spain; Biocomputation and Complex Systems Physics Institute (BIFI)-Joint Unit GBsC-CSIC, University of Zaragoza, 50018 Zaragoza, Spain; Departamento de Bioquímica y Biología Molecular y Celular, Facultad de Ciencias, University of Zaragoza, 50009 Zaragoza, Spain; Biocomputation and Complex Systems Physics Institute (BIFI)-Joint Unit GBsC-CSIC, University of Zaragoza, 50018 Zaragoza, Spain; Departamento de Bioquímica y Biología Molecular y Celular, Facultad de Ciencias, University of Zaragoza, 50009 Zaragoza, Spain; Aragon Health Research Institute (IIS Aragón), 50009 Zaragoza, Spain

## Abstract

**Motivation:**

The stability of protein interfaces influences protein dynamics and unfolding cooperativity. Although in some cases the dynamics of proteins can be deduced from their topology, much of the stability of an interface is related to the complementarity of the interacting parts. It is also important to note that proteins that display non-cooperative unfolding cannot be rationally stabilized unless the regions that unfold first are known. Being able to identify protein interfaces that are significantly less stable would contribute to our understanding of protein dynamics and be very valuable in guiding the rational stabilization of proteins with non-two-state unfolding equilibria.

**Results:**

We introduce *ProteinLIPs*, a web server that detects interfaces of high polarity and low packing density, termed LIPs. Each LIP consist of a continuous sequence segment (mLIP) plus its contacting residues (cLIP). *ProteinLIPs* scans monomeric and oligomeric proteins and provides graphical sequence profiles and interactive 3D visualizations of the detected LIPs. Statistical analysis of 53 protein domains from 10 superfamilies shows the two parts of a LIP present distinct characteristics. mLIPs are conserved, structurally unstable and enriched in polar residues, whereas cLIPs are more stable, less conserved, and enriched in apolar residues. Besides, cLIPs are enriched in small-molecule binding site residues, suggesting they play a role in ligand interaction, likely facilitated by instability of the associated mLIPs. *ProteinLIPs* provides a user-friendly platform for the automated identification and visualization of LIPs and can be used to guide the engineering of non-two-state proteins where LIPs constitute preferential targets for thermostabilization.

**Availability and implementation:**

**ProteinLIPs** is publicly available at https://lips.bifi.es/.

## 1 Introduction

The biological function of folded proteins depends on both their structure and intrinsic dynamics (Medina *et al.* 2021). Protein cores tend to concentrate apolar residues and to be well packed (Baldwin and Matthews 1994), although some degree of heterogeneity in them is expected. We reported that continuous protein stretches involved in protein folding dynamics or exhibiting low local stability tend to form interfaces characterized by higher polarity and lower packing density than average (Espinosa-Angarica and Sancho 2012). In one protein, *Anabaena PCC 7119* apoflavodoxin (Sancho 2006), for which detailed structural and thermodynamic data were available, such interfaces were observed to unfold at lower temperatures than the rest of the protein (Campos *et al.* 2004a). To refer to protein interfaces exhibiting a high polarity and a low packing density, we coined the term LIP: Light Interface of high Polarity (Espinosa-Angarica and Sancho 2012). As interfaces, LIPs comprise two components: the ‘main’ part (mLIP) consists of a continuous stretch of amino acid residues that meet the polarity and packing criteria detailed in the Section 2, while the ‘counterpart’ (cLIP) comprises the residues—not necessarily forming a continuous sequence—in contact with the main part. To facilitate the identification of LIPs in proteins, we have developed *ProteinLIPs* (https://lips.bifi.es/), a web server that analyses PDB files in search of LIPs and displays their location within both the sequence and the 3D structure. For oligomeric proteins, *ProteinLIPs* identifies the LIPs of each monomer as well as those formed between contacting ones. Statistical analysis of LIP composition, stability and conservation in monomeric proteins suggests a dynamic role for mLIPs and a stabilizing counterpart function for cLIPs. *ProteinLIPs* can help detect highly dynamical regions potentially involved in protein function. Moreover, LIPs represent instability hotspots that, once identified, may guide rational stabilization strategies enabling a more successful biotechnological exploitation of complex proteins.

## 2 Materials and methods

### 2.1 Algorithm for calculation of mLIPs in monomers

**Figure 1.**
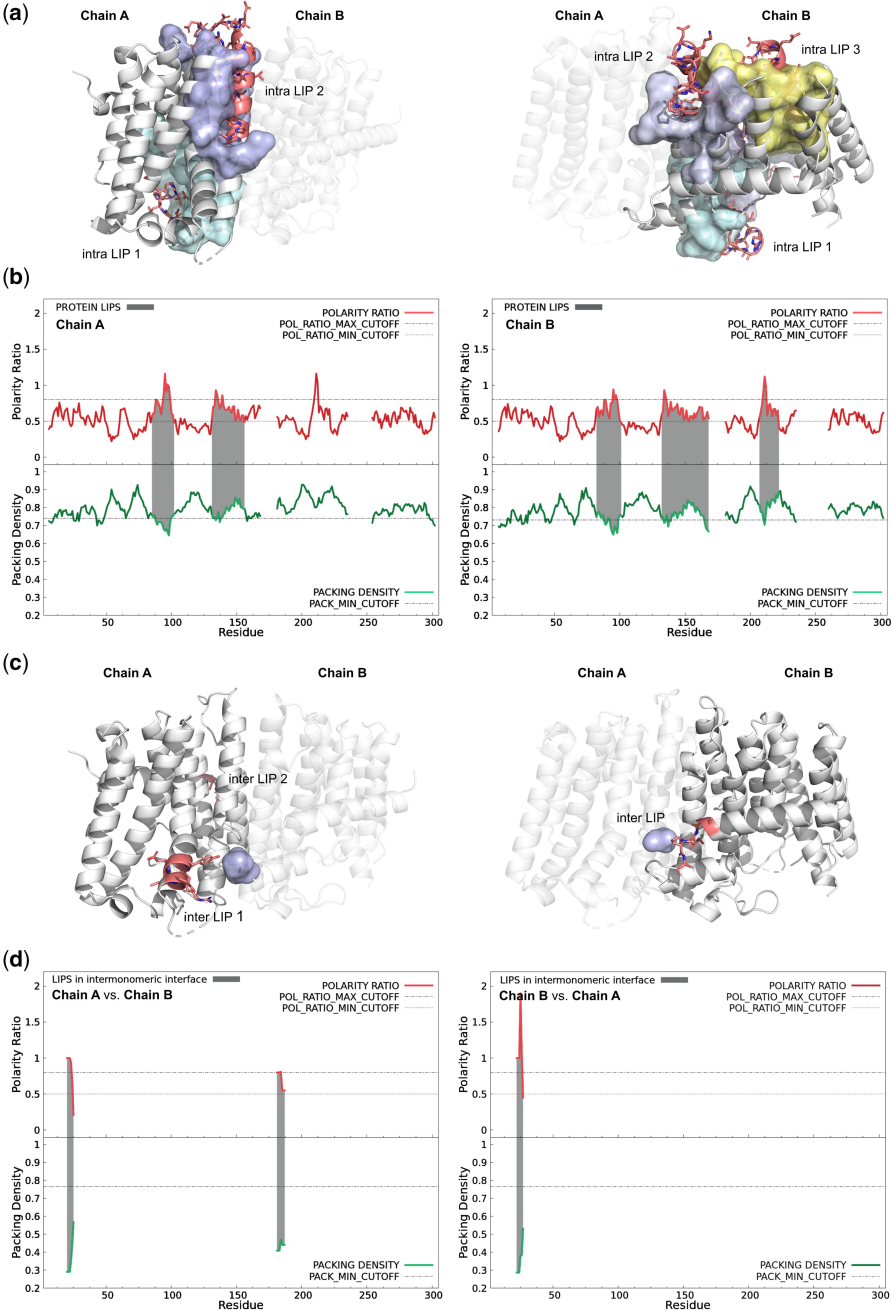
Representations of computed LIPs and the associated sequence profiles for the homo-dimer crystal structure of geranylgeranyl diphosphate synthase 1 from *Oryza sativa* (PDB ID: 5XN5). (a) Depiction of the computed intra-mLIPs (red-coloured ribbon + sticks) and their interacting counterparts (surface; cLIP-1 in cyan, cLIP-2 in pale blue, and cLIP-3 in yellow) for monomer A (left) and monomer B (right), respectively. The opposing chain in the dimer is also depicted (cartoon) but with higher transparency. Note that, although some residues within the LIPs may appear partially exposed, the interfaces involved in the packing of an mLIP against its corresponding cLIP are always buried; (b) top-bottom dual sequence profiles―with calculated PR at the top (red thick line) and calculated ρ at the bottom (green thick line)―showing the computed intra-mLIPs (grey-shadowed regions) for monomer A (left) and monomer B (right). In both the top PR and the bottom ρ panels, horizontal black dashed lines indicate the selected cut-offs used for the calculation of the mLIPs (PR minimum of 0.8 for peaks and 0.5 for the baseline at the top plot, and the mean ρ minus one standard deviation at the bottom plot), as described (Espinosa-Angarica and Sancho 2012). Gaps found in the protein structure (non-solved residues) appear as unconnected regions in the PR and ρ profiles; (c) depiction of the computed inter-mLIPs (red-coloured ribbon + sticks) and their interacting cLIPs (in pale blue) for interface A: B (left) and interface B: A (right), respectively; (d) top-bottom dual sequence profiles showing the computed inter-mLIPs (grey-shadowed regions) for interaction A: B (left) and interaction B: A (right). Profiles’ colours and appearance, as well as the values at horizontal dashed lines (cut-offs used in the mLIP calculation) are identical to those described for panel b.

Protein LIPs are interfaces formed between a continuous protein segment—referred to as the mLIP—and the residues on which it packs—collectively termed the cLIP. The algorithm for calculating mLIPs of protein monomers (‘intra-mLIPs’, Fig. 1a) was described in detail in previous work (Espinosa-Angarica and Sancho 2012). It is based on per-residue estimations of two physical parameters of interfaces in the queried protein: the polarity ratio (PR) and the packing density (ρ). The polarity ratio is defined as:


(1) $$\mathrm{PR}=\frac{\sum_{i=1}^{m}{\mathrm{SASA}}_{{\left(\mathrm{polar}\right)}_{i}}}{\sum_{j=1}^{n}{\mathrm{SASA}}_{{\left(\mathrm{apolar}\right)}_{j}}}$$

where the solvent-accessible surface areas (SASA) per atom in the interfaces are estimated using NACCESS v2.1.1 (Hubbard and Thornton 1993) with a spherical probe of radius 1.4 Å.

The packing density ρ is computed as:


(2) $$\rho =\frac{\sum_{i=1}^{N}{V^{o}}_{i}}{\sum_{i=1}^{N}V_{i}}$$

where the numerator corresponds to Voronoi standard atomic volumes (Tsai *et al.* 1999) and the denominator to the actual Voronoi atomic volumes of the atoms at the interface, as calculated by CALC-VOL (Voss and Gerstein 2005). These two parameters are iteratively calculated using a sliding probe of eight contiguous residues with the resulting value assigned to the fourth one. After scanning the entire protein chain, a dual sequence profile is constructed, with PR values plotted at the top (red thick line) and ρ values at the bottom (green thick line; see Fig. 1b). Intra-mLIPs are then identified as regions that simultaneously satisfy the established PR and ρ thresholds (Espinosa-Angarica and Sancho 2012). Specifically, these regions correspond to PR peaks with maximum values above 0.8, which extend on both sides of the peak to include adjacent residues with PR values above the baseline of 0.5. The regions should also include at least one residue with ρ values below the mean ρ minus one standard deviation (calculated across the entire profile) (Espinosa-Angarica and Sancho 2012).

The *ProteinLIPs* server can process both protein monomers and oligomers. It calculates the mLIPs present in each monomer, as well as those formed at monomer–monomer interfaces (inter-mLIPs). For homo- or heterodimers, four separate calculations are performed: one for intra-mLIPs in chain A, one for those in chain B, and two additional ones for inter-LIPs at the A: B interface, probed independently from chains A and B (see the next section and Fig. 1). For trimers or higher-order oligomers, users can specify the monomer pair to be analysed for intra- and inter-mLIPs. This comprehensive approach has been adopted after examining mLIPs in a dataset of 50 protein dimers—including symmetric and asymmetric biological units—which revealed that mLIP profiles frequently differed between chains A and B, both for intra- and inter-mLIPs. This is illustrated in Fig. 1 by the mLIP profiles (panels b and d) computed for the homodimeric geranylgeranyl diphosphate synthase 1 from *Oryza sativa* (GGPPS, PDB ID 5XN5). It is important to note that the intra-mLIP profiles and their corresponding cLIPs obtained from homodimers with structurally identical subunits are identical (data not shown), unless the two chains differ in missing residues. In contrast, the inter-mLIPs may differ slightly if the monomer–monomer interaction is not a perfect mirror image between the two subunits.

### 2.2 Calculation of mLIPs in monomer–monomer interfaces of oligomers

The algorithm implemented for the calculation of inter-mLIPs mirrors that used for intra-mLIPs, with one notable difference. For intra-mLIPs, the residues interacting with the eight-residue probe are searched within the remaining fragment of the monomer once the probe is cropped out. For inter-mLIPs, the sliding probe from, e.g. monomer A searches for interacting residues on the entirety of monomer B. Once the probe reaches the end of monomer A, an analogous calculation is performed after swapping the roles of the two chains. Panel c in Fig. 1 shows structural representations of the inter-mLIPs of the homodimeric GGPPS protein, obtained from the profiles in panel d.

### 2.3 Calculation of cLIPs

The set of residues packed against an intra- or inter-mLIP is here referred to as a cLIP. The residues composing a cLIP are identified using an ad-hoc algorithm that analyses the per-residue change in SASA in the protein as a result of having cropped out the corresponding mLIP: ΔSASA = SASA_i (full-protein)_ – SASA_i (cropped-protein)_, where *i* refers to the residue indices. Specifically, residues exhibiting a ΔSASA > 1.0 Å^2^ are considered part of the cLIP associated with the removed mLIP.

### 2.4 Server implementation

**Figure 2.**
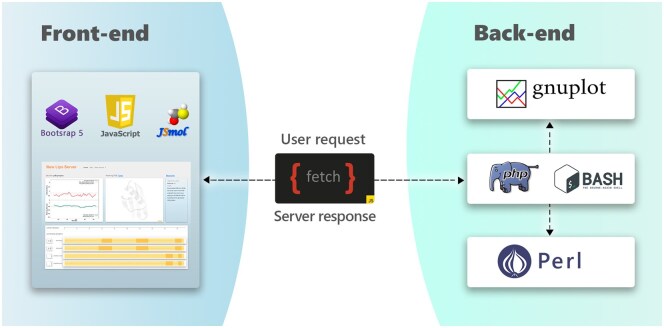
*ProteinLIPs* server’s scheme. Overview of the web server implementation and the tools involved.

A general scheme of the server implementation is shown in Fig. 2. ProteinLIPs uses Bootstrap version 5.2.3 (Bootstrap, San Francisco, CA) for client-side (front-end) presentation. API Fetch and PHP (version 8.1, Coretechs, Kensington, MD) are used to handle user requests on the server side. Upon a PDB ID query, a Bash script connects—in the back-end—to the PISA server (Krissinel and Henrick 2005, 2007) or, if the structure is not found there, to the Protein Data Bank API to download the PDB file of the biological unit and associated metadata. Alternatively, users can upload a PDB-formatted protein structure file, which will be considered to be the biological unit. From the requested or uploaded PDB file, a custom Bash script that integrates Perl (version 5) and Gnuplot (version 5.4) sub-scripts into its workflow calculates the mLIP and cLIP components of all LIPs found in the protein. The open-source JavaScript viewer JSmol (version 16.2.7) is integrated into the server to visualize the calculated LIPs on the 3D structure of the protein.

The minimum browser versions compatible with the server across common operating systems are listed in Table 1, available as supplementary data at *Bioinformatics* online. An updated browser is recommended, and JavaScript must be enabled.

### 2.5 Protein superfamilies and structural domains selected for analysis

A set of 50 representative domains/proteins from 10 different folding superfamilies (5 domains per superfamily, Table 2, available as supplementary data at *Bioinformatics* online) was initially selected from the CATH database (Knudsen and Wiuf 2010) to carry out structural and compositional analyses of LIPs. The selection covers the 3 main folding classes in CATH: mainly-alpha (α), mainly-beta (β), and alpha-beta (αβ). No distinction between α/β and α + β classes is made in CATH. Domain assignments to class were done by earlier versions considering α/β and α + β as different classes, but those assignments showed there was considerable overlap between them and it was decided they would be more naturally represented as a single αβ class (Michie *et al.* 1996). The distribution of selected superfamilies per folding class was: 3 from class α, 3 from class β, and 4 from class αβ. The selection of protein architecture, fold and superfamily within these classes followed an abundance criterion. A superfamily was deemed suitable if it contained five or more domains, each one between 50 and 250 residues in length, without bound ligands, and not containing domain repeats. A preliminary analysis indicated that 3 of the initially selected 50 proteins/domains lacked LIPs (Table 3, available as supplementary data at *Bioinformatics* online). Therefore, for the composition and enrichment analyses shown below, these were replaced with 3 domains from the same superfamilies as the original ones. For evolutionary conservation and stability analyses, a dataset of 53 proteins/domains was used, including the original 50 and the 3 replacements.

### 2.6 Calculation of evolutionary conservation scores (Consurf) and stability profiles (SWOTein)

The 53 domains listed in Tables 2 and 3, available as supplementary data at *Bioinformatics* online were analysed using the Consurf (Ben Chorin *et al.* 2020) and SWOTein (Hou *et al.* 2021) servers to obtain evolutionary conservation scores and stability profiles, respectively. Default parameters shown in Table 4, available as supplementary data at *Bioinformatics* online were used for Consurf. SWOTein has no adjustable parameters. The metrics from these tools were compared within and outside the following structural features: (i) mLIPs, (ii) cLIPs, (iii) full LIPs (calculated by ProteinLIPs), (iv) alpha helices [calculated as ‘H’ by DSSP (Kabsch and Sander 1983, Touw *et al.* 2015)], (v) beta sheets (calculated as ‘B’ or ‘E’ by DSSP), and (vi) coil structures (reported as blanks by DSSP). We deliberately excluded 3_10_ and Π helix DSSP categories from our alpha category, and turns and bends from our random coil category, as their abundances are low and considering them might introduce spurious variability in the analysis. Comparisons were made using a Student’s *t*-test (Student 1908) for mean differences and a point-biserial correlation analysis. Effect sizes were estimated using Cohen’s d statistic on mean differences (Cohen 1988).

### 2.7 LIPs composition and enrichment analyses

The amino acid composition of LIPs (and their mLIP and cLIP components) was analysed in a set of 50 proteins/domains from CATH (Knudsen and Wiuf 2010) (Table 2, available as supplementary data at *Bioinformatics* online). The sequences used were those from the processed domains in the PDB files. A LIP enrichment factor (EF) was calculated as the ratio of relative frequencies in LIP and NO-LIP regions:


(3) $$\mathrm{EF}=\frac{\upsilon (\mathrm{LIP})}{\upsilon (\mathrm{NO}-\mathrm{LIP})}$$

for each of the 20 proteinogenic amino acids and for the following groups: non-polar (A, G, V, L, I, P, M, C, F, W), polar uncharged (Y, S, T, N, Q), polar negatively charged (D. R), and polar positively charged (K, R, H).

### 2.8 LIPs enrichment analyses in small molecule binding sites

To analyse the potential relationship between LIPs and functional sites, SITE annotations from the PDB files of the 50 proteins/domains (Table 2, available as supplementary data at *Bioinformatics* online) were extracted (Table 5, available as supplementary data at *Bioinformatics* online). As informed in the PDB files, all SITE annotations were made by software. The proportion of LIP-located residues was calculated both for SITE-labelled residues and for all residues of the domain (as a control). The statistical significance of SITE enrichment in LIPs was assessed using a two-tailed Z-test comparing LIPs proportions between SITE and full domain residues. For analyzing the exposure of the residues annotated as SITEs, a classification in buried (relative exposure lower than 10%), intermediate exposure (relative exposure between 10 and 40%) and exposed residues (relative exposure over 40%) has been made. For the calculation of their relative exposure, the maximum exposure values for each type of residue obtained by Tien *et al.* (Tien *et al.* 2013) have been used.

## 3 Results and discussion

### 3.1 Server description and functionalities

LIPs are formed by two interacting surfaces—mLIPs and cLIPs—that exhibit high overall polarity and are poorly packed against each other (Espinosa-Angarica and Sancho 2012). To facilitate their identification in protein structures, we have developed *ProteinLIPs*, an online server that performs automated 3D-structure analysis of PDB files. *ProteinLIPs* can analyse protein monomers and oligomers, calculating intra-mLIPs for each monomer as well as inter-mLIPs located at monomer monomer interfaces. Upon submission of a query protein, the server generates top-bottom LIP profiles alongside the protein sequence (with PR on top and ρ on the bottom, see Fig. 1b and d). These profiles are complemented by a 3D molecular visualization using the integrated JavaScript viewer JSmol, which displays the mLIPs on the protein structure along with their interacting cLIPs (Fig. 1a and c). To enhance usability, expandable sequence bars implemented via the RCSB Saguaro 1D Feature Viewer (Segura *et al.* 2021) provide a detailed view of the mLIP locations along the sequence. Highlighted mLIPs act as toogle buttons enabling users to easily show or hide the corresponding LIP on the structure within the JSmol panel. *ProteinLIPs* retains the original residue numbering from the processed PDB files. Any gaps in residue numbering are reflected in the sequence profiles and annotated in the legend. Structural gaps (non-solved residues) appear as discontinuities in the LIP profiles (Fig. 1a). Once the calculation is complete, all analysis results are displayed through the server interface. If the user has opted in, a link to the results page is also sent via email. The results screen includes a download button for a compressed ZIP file containing the calculated sequence profiles in PNG format, the PDB structure file used in the analysis, and a summary report. The summary includes the spans of the identified intra-mLIPs and inter-mLIPs (if applicable), the interacting residues comprising each associated cLIPs, and information on any structural or sequence gap as well as the annotated SITEs found in the queried PDB entry.

### 3.2 Conservation of LIPs: statistical analyses from Consurf score

A high conservation of polarity and packing density profiles—and thus of LIPs—was previously observed among a few proteins belonging to three folding superfamilies and, to a lesser extent, among proteins in different superfamilies of the same folding class (Espinosa-Angarica and Sancho 2012). Our current analysis, based on a dataset of 50 protein domains (Table 2, available as supplementary data at *Bioinformatics* online), confirms the presence of LIPs across all 10 protein superfamilies analysed. We hypothesize that, in monomeric proteins, LIPs are functionally relevant and thus expected to be conserved within protein families. To test this, we have performed a statistical analysis using per-residue evolutionary conservation scores obtained from Consurf (Ben Chorin *et al.* 2020), which calculates conservation based on multiple sequence alignments of homologous proteins. Consurf scores were obtained for 53 domains selected from CATH (Knudsen and Wiuf 2010) (see Section 2, Tables 2 and 3, available as supplementary data at *Bioinformatics* online). Our analysis has focused on comparing the mean conservation scores of residues within specific structure elements to those of residues outside those elements. Thus, the mean of the conservation score has been calculated for all residues in a given structure element (e.g. the mean of all residues located in mLIPs) and the mean of the residues outside of such element (e.g. the mean of all residues not located in mLIPs) has been subtracted in order to obtain the mean difference. In this context, a positive mean difference indicates greater sequence conservation within the structure element, while a negative value suggests lower sequence conservation. As a reference for comparison, conservation scores and mean differences for alpha helices, beta sheets and random coil regions have been obtained. The results of the mean difference analysis (Table 1) show that evolutionary conservation is significantly higher at mLIPs (+0.139) and lower at cLIPs (–0.182) and beta conformations (−0.278). In contrast, no significant conservation is observed for full LIPs (–0.042), alpha helices (+0.039) or coil (+0.013) regions. These findings are supported by the point-biserial correlation analysis summarized in Table 6, available as supplementary data at *Bioinformatics* online. Additionally, the effect sizes computed using Cohen’s d (Table 7, available as supplementary data at *Bioinformatics* online) suggest that the statistical significance of these results is not an artifact of the large dataset size (total number of residues analysed = 7103).

**Table 1.** Mean difference analyses for LIPs and secondary structure elements based on Consurf (evolutionary conservation) and SWOTein (stability) per-residue scores.^a^

Structure element	Consurf Score^b^	SWOTein Score (All)^c^
mLIPs^d^	0.139^g^	0.688^g^
cLIPs^d^	‒0.182^g^	‒0.199
Full LIPs^e^	‒0.042	0.227
Alpha^f^	0.039	‒1.326^g^
Beta^f^	‒0.278^g^	0.171
Coil^f^	0.013	1.202^g^

a Mean differences between scores calculated for residues inside and outside a given structural element.

b Positive mean differences indicate higher sequence conservation of residues in a structure element compared to the rest of the protein. Negative mean differences suggest lower sequence conservation.

c Original stability-related function values provided by SWOTein are positive for destabilizing contributions to global stability and negative for stabilizing contributions. To maintain this convention here, positive mean differences indicate a destabilizing effect of the structure element, whereas negative mean differences suggest a stabilizing effect. ‘All’ stands for the sum of SWOTein prediction values for ‘Distance’, ‘Accessibility’ and ‘Torsion’ stability components.

d mLIPs and cLIPs residues as calculated by the *ProteinLIPs* server.

e Full LIPs encompass residues in mLIPs or cLIPs.

f Per-residue assignments of secondary structure as calculated by the DSSP program (Kabsch and Sander 1983).

g $P_{\mathrm{value}}<0.005$ using two tailed Student’s *t*-test for mean differences without multiple test correction.

### 3.3 Stability of LIPs: statistical analyses from SWOTein metrics

In previous work (Espinosa-Angarica and Sancho 2012), we illustrated a qualitative correlation between LIPs and conformationally unstable regions in a limited set of proteins for which diverse stability data had been reported. To quantitatively assess whether LIPs tend to be structurally unstable, we have conducted a statistical analysis using per-residue stability metrics calculated by SWOTein (Hou *et al.* 2021). For that, per-residue SWOTein scores were obtained for 53 protein domains from CATH (Knudsen and Wiuf 2010) (see Section 2 and Tables 2 and 3, available as supplementary data at *Bioinformatics* online). The original SWOTein metrics assign positive values to residues contributing unfavourably to global stability (i.e. destabilizing) and negative values to residues that contribute favourably (i.e. stabilizing). Preserving this convention, a positive mean difference in our analysis indicates that the structure element is relatively destabilizing, whereas a negative mean difference indicates a stabilizing effect. As shown in Table 1, mLIPs (+0.688) and random coil regions (+1.202) are significantly less stable than the other analyzed protein elements, while beta regions (+0.171) exhibit near-neutral stability. Conversely, alpha helices are more stable (–1.326), which aligns well with previously reported findings (Abrusán and Marsh 2016). Residues at cLIPs appear to exhibit a mild stabilizing effect (–0.199), but the difference is not statistically significant. For full LIPs, the opposing contributions of mLIPs and cLIPs yield a non-significant destabilizing mean difference of +0.227. Effect sizes calculated using Cohen’s d (Table 7, available as supplementary data at *Bioinformatics* online) indicate that the observed significance for the destabilizing nature of mLIPs is not simply a result of the large sample size. As it seems, mLIPs are evolutionary conserved and yet they are conformationally unstable regions. As such, they seem well-suited to participate in protein dynamics associated with protein folding and functional transitions.

### 3.4 Per-residue composition of LIPs and enrichment analyses

**Figure 3.**
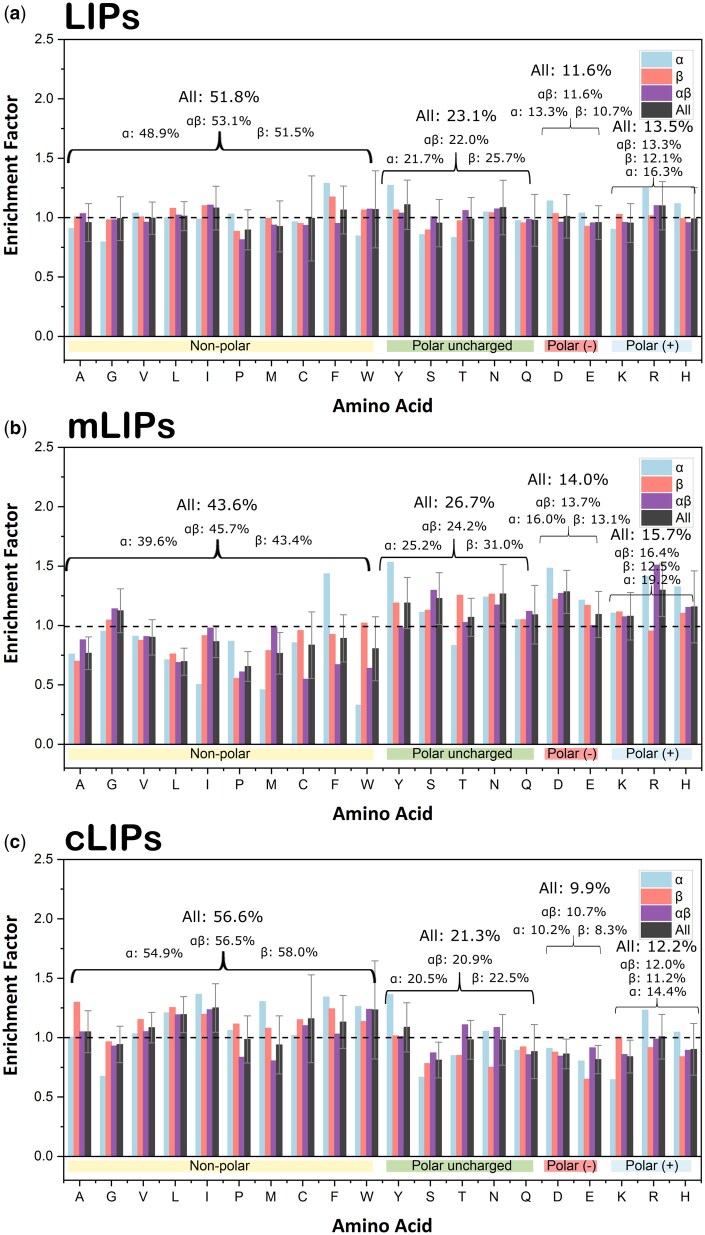
LIPs enrichment and composition analysis on the set of 50 proteins/domains collected from CATH (Knudsen and Wiuf 2010). Vertical bars represent per amino acid enrichment factors (*y*-axis), calculated as the ratio of relative frequencies (e.g. υ(LIPs)/υ(NO-LIPs)), across each CATH folding class (α, β, and αβ) and all analysed proteins (all). Distributions (in %) of amino acid types (polar, polar uncharged, positively charged and negatively charged) per folding class are given on top of the bars. Data obtained for: (a) whole LIPs; (b) mLIPs; and (c) cLIPs. The legend in the top-right corner of each graph indicates the colours representing the folding classes. For clarity, error bars (standard errors) are represented only on the residue bars corresponding to ‘all’ the folding classes. Coloured horizontal bars below the *x*-axis display the polarity group to which each amino acid belongs to: pale yellow for non-polar, green for polar uncharged, red for positively charged, and pale blue for negatively charged.

Given the evolutionary conservation of protein LIPs and their potential functional relevance, we have conducted a detailed analysis of their amino acid composition—along with that of their mLIP and cLIP components—across the 50 domains listed in Table 2, available as supplementary data at *Bioinformatics* online. The frequency distributions of each amino acid and amino acid type in LIP and non-LIP regions, classified by CATH folding classes, are presented in Fig. 1, available as supplementary data at *Bioinformatics* online. Based on these distributions, a per-residue enrichment analysis has been performed (Fig. 3). Considering all proteins together, full LIPs do not exhibit any clear pattern of residue enrichment (Fig. 3a), although there is notable variability within the alpha class (F, Y, D, R: EF > 1, and G, W, S, T: EF < 1) compared to the other protein classes. A clear pattern emerges, however, when the analysis is separated by LIP components. mLIPs are significantly enriched in polar residues—specifically, Y, S, N, D, and R (Fig. 3c)—while cLIPs are significantly enriched in non-polar ones (L, I, and W; Fig. 3d). The overall composition of LIPs, mLIPs, and cLIPs, expressed as percentage of residue types, is as follows: non-polar (51.8, 43.6 and 56.6), polar neutral (23.1, 26.7, and 21.3), negatively charged (11.6, 14.0, and 9.9), and positively charged (13.5, 15.7, and 12.2). This enrichment pattern is consistent among the three folding classes analysed (α, β, and α + β; Fig. 3). Thus, while LIPs as a whole are more polar than typical protein interfaces, this characteristic primarily arises from the high polarity of mLIPs, with cLIPs being relatively enriched in non-polar residues. The contrasting polarity between the two LIP components likely contributes to their poor internal packing. This compositional pattern may also explain the calculated instability of mLIPs, which are rich in polar, buried residues, and the stability of cLIPs, which are enriched in apolar ones.

### 3.5 LIPs enrichment analyses in small molecule binding sites

To further explore the potential functional relevance of LIPs, we have assessed their association with annotated SITE residues—a proxy for functional regions—across the 53 PDB files analysed (Table 2). Residues labelled as SITE were found to be significantly enriched (for a confidence of 95%) within LIPs (65% versus 52% in the full domain) cLIPs (47% versus 34%), and mLIPs (32% versus 25%) (see Table 2). As all the annotated SITEs in the analyzed dataset (Table 5, available as supplementary data at *Bioinformatics* online) correspond to small ligand binding sites, our analysis suggests that cLIPs may be involved in the binding of small ligands. For that, the relative instability of their mLIP counterparts may facilitate ligand access to the binding site. As no protein–protein interaction sites were annotated in the dataset, we are currently unable to assess whether LIPs are similarly involved in protein–protein interactions—an involvement we consider probable.

**Table 2.** Residues contained in LIPs and exposure for SITE-annotated residues and for the full domains.

Structure element	SITEs^a^		Domains^b^		
	No. of residues	p^c^ (%)	No. of residues	p^c^ (%)	*P*-value^d^
mLIPs^e^	95	31.6%	1915	25.0%	**0.010**
cLIPs^e^	143	47.5%	2635	34.4%	**3×10^–6^**
Full LIPs^f^	196	65.1%	3983	51.9%	**7×10^–6^**
NO LIPs^g^	105	34.9%	3685	48.1%	**7×10^–6^**
Buried^h^^,^^i^	113	37.5%	3074	40.0%	0.385
Intermediate^h^^,^^j^	130	43.2%	2679	34.9%	**0.003**
Exposed^h^^,^^k^	57	18.9%	1907	24.8%	**0.020**

a Residues annotated as SITE in the original PDB of the 50 analysed domains (Table 2, available as supplementary data at *Bioinformatics* online).

b Residues in the 50 analysed domains (Table 2, available as supplementary data at *Bioinformatics* online).

c Calculated as the percentage of residues in each category out of the total number of analysed residues, the sum of LIPs and NO LIPs.

d *P*-value calculated using a two-tailed *Z*-test for difference of proportions. Values in bold denote statistical significance at the 95% confidence level.

e mLIPs and cLIPs residues as calculated by the *ProteinLIPs* server.

f LIP residues conforming an mLIP or a cLIP.

g Residues not present in an mLIP nor a cLIP.

h Relative exposure could not be calculated for non-canonical amino acids, and therefore the sum of analysed residues for this property (300) differs from the sum of residues classified as LIPs or NO LIPs (301).

i Relative exposure under 10%.

j Relative exposure between 10% and 40%.

k Relative exposure over 40%.

In the analyzed structures (domains), a residue can often be classified both as part of an mLIP and as a cLIP corresponding to another mLIP. However, each residue is counted only once as part of an LIP. This is why the total number of residues in LIPs does not match the sum of residues in mLIPs and cLIPs.

We have checked if the observed enrichment of LIPs in SITE residues is related to a common differential solvent exposure of LIP and SITE residues. Firstly, we have compared the exposure of SITE residues relative to that of all the residues of a given domain, considering three exposure levels (Buried, Intermediate, and Exposed) (lower part of Table 2). A significant change in proportion is observed for Intermediate and Exposed residues, which are, respectively, more and less abundant in SITES. Thus, a preference towards residues with intermediate exposure and against fully exposed residues is observed in SITEs, reflecting that a SITE must provide some ordered environment for interaction. Secondly, we have analysed whether SITE residues in LIPs and out of them share a common pattern of solvent exposure. For that we have disaggregated SITE residues according to their location and the exposure profile has been obtained for SITE residues in the different LIP components and outside LIPs (Table 3). The SITE and full domain exposures reported in Table 2 have been added to Table 3 for easier comparison. The exposure profiles for SITE residues in cLIPs, mLIPs and full LIPs are similar but clearly different from that of SITE residues outside LIPs (NO LIPs). Comparing full LIPs to NO LIPs, a significantly higher proportion of buried residues (42.3% versus 28.8%) is observed (*P*-value of .022), while a *P*-value of 0.053 is obtained for the lower proportion of exposed ones (15.8% versus 25.0%). No significant differences were found with a 95% confidence interval using a Z-test between LIPs (either mLIPs, cLIPs or full LIPs) and SITE residues. However, this may be due to the low number of residues when subdividing the categories and to the fact that over 65% of the SITE residues are in LIPs, so a qualitative comparison is performed. Compared to the SITE profiles, the LIP profiles in Table 3 show a higher proportion of buried residues and a lower proportion of exposed ones. As a whole, buried residues are the majority class in both LIPs (being significantly higher than NO LIPs) and full domains, unlike in SITEs, where intermediate exposure residues are the most frequent class. This difference between LIPs and SITEs profiles deems unlikely the possibility that the enrichment of LIPs in SITE residues is related to a common differential exposure relative to full domains.

**Table 3.** Exposure profile of SITE-annotated residues by LIP component.

Structure element	Buried^a^		Intermediate^b^		Exposed^c^		
	No. of residues	p^d^ (%)	No. of residues	p^d^ (%)	No. of residues	p^d^ (%)	Total no. of residues^e^
cLIPs^f^	66	45.0	55	39.3	22	15.7	143
mLIPs^f^	43	43.5	37	40.2	15	16.3	95
Full LIPs^g^	83	42.3	82	41.8	31	15.8	196
No LIPs^h^	30	28.8	48	46.2	26	25.0	104
SITEs^i^	113	37.5	130	43.2	57	18.9	301
Domains^i^	3074	40.0	2679	34.9	1907	24.8	7677

a Relative exposure under 10%.

b Relative exposure between 10% and 40%.

c Relative exposure over 40%.

d The percentage of residues with a certain level of exposure is calculated as the number of residues in a structure element with a certain level of exposure out of the total number of residues for such element.

e Relative exposure could not be calculated for non-canonical amino acids, and therefore the sum of no. of residues may be lower than the total no. of residues, and the proportions may not sum up to 100%.

f mLIPs and cLIPs residues as calculated by the *ProteinLIPs* server.

g LIPs defined as residues in an mLIP or a cLIP.

h Residues not present in an mLIP nor a cLIP.

i Taken from Table 2 for the sake of comparison.

### 3.6 Anatomy of LIPs as dynamic regions and their potential as targets for thermostabilization

LIPs are interfaces formed by a continuous sequence segment (the mLIP) and a set of facing residues (cLIP). These two parts differ markedly in both sequence composition and evolutionary characteristics. The mLIP is enriched in destabilizing residues but displays a significant degree of evolutionary conservation. This combination is reminiscent of enzyme active site residues (Shoichet *et al.* 1995, Siddiqui 2017), suggesting that mLIPs may play functional roles, potentially acting as dynamic segments capable of undergoing local conformational changes or partial unfolding to support protein activity. In contrast, cLIPs are enriched in stabilizing residues and may play a complementary role maintaining local structural integrity in the vicinity of the less stable mLIPs. This architectural arrangement parallels recent observations in enzyme catalytic sites, where conserved, destabilizing residues at the catalytic core are surrounded by non-conserved, stabilizing ones (Hou *et al.* 2023). Moreover, our findings suggest a potential involvement of LIPs in small ligand binding. Even the cLIP parts, that are not evolutionarily conserved and are therefore unlikely to constitute complete binding sites on their own, seem to contribute to such sites. The lower stability of their adjacent mLIPs may facilitate ligand access.

Importantly—with the caveat that modifying a LIP could in some cases affect protein activity— we note that LIPs are preferential targets for protein stability engineering in non-two-state proteins. Due to their low local stability, LIPs are likely among the first regions to unfold during the non-cooperative transitions characteristic of such proteins. Consequently, LIP stabilization offers a direct and effective strategy to increase their relevant stability (Sancho *et al.* 2002, Campos *et al.* 2004b). This approach has been convincingly demonstrated in apoflavodoxin, a three-state protein (Sancho 2006). Targeted stabilization of its main LIP increased the cooperativity of its thermal unfolding—effectively converting it into a two-state protein—and greatly improved its thermostability, without compromising its electron transfer function (Lamazares *et al.* 2017).

## Supplementary Material

btaf499_Supplementary_Data

## Data Availability

**ProteinLIPs **is publicly available at https://lips.bifi.es/
