# Supplementary material for: ProteinLIPs: a web server for identifying highly polar and poorly packed interfaces in proteins
Source: Bioinformatics. 2025 Sep 10;41(9):btaf499. doi: 10.1093/bioinformatics/btaf499 (PMC12457736; doi:10.1093/bioinformatics/btaf499)
Supplement: btaf499_Supplementary_Data [file btaf499_supplementary_data.zip › García-Cebollada_et_al_(Suppl._Material) - revised.docx]

| **SUPPORTING INFORMATION**  ProteinLIPs: a web server for identifying highly polar and poorly packed buried interfaces in proteins  Helena García-Cebollada^1,2^, Alfonso López^1^, Vladimir E. Angarica^1,2ǂ^, Juan José Galano-Frutos^1,2†*^ and Javier Sancho^1,2,3*^  ^1^Biocomputation and Complex Systems Physics Institute (BIFI)-Joint Unit GBsC-CSIC, University of Zaragoza, 50018 Zaragoza, Spain. ^2^Departamento de Bioquímica y Biología Molecular y Celular, Facultad de Ciencias, University of Zaragoza, 50009 Zaragoza, Spain. ^3^Aragon Health Research Institute (IIS Aragón), 50009 Zaragoza, Spain. ^ǂ^Present address: Center for Computational Biology, DUKE-NUS Medical School, Singapore. ^†^Present address: Certest Biotec S.L., 50840 San Mateo de Gallego, Zaragoza, Spain.  *To whom correspondence should be addressed:  Juan José Galano-Frutos (ORCID: 0000-0002-1896-7805): [juanjogf@unizar.es](mailto:juanjogf@unizar.es)  or  Javier Sancho (ORCID: 0000-0002-2879-9200): [jsancho@unizar.es](mailto:jsancho@unizar.es) |
| --- |

**CONTENT**

**SI TABLES** 3

SI Table 1. *ProteinLIPs*’ browsers compatibility 3

SI Table 2. Data related to the 50 protein domains from 10 CATH superfamilies used for the LIPs composition and enrichment analyses. 4

SI Table 3. Data related to protein domains from the initial dataset predicted to lack LIPs. 8

SI Table 4. Default parameters for Consurf calculations. 9

SI Table 5. Binding sites annotated in the analysed domains. 10

SI Table 6. Point-biserial correlation analysis for LIPs and secondary structure elements from Consurf (evolutionary conservation) and SWOTein (stability) per-residue scores. 13

SI Table 7. Effect size analysis (Cohen’s d) of the presence of LIPs and secondary structure elements in the selected protein set from Consurf (evolutionary conservation) and SWOTein (stability) per-residue scores.. 14

**SI FIGURE** 15

SI Figure 1. Per amino acid frequency in LIPs regions across all the CATH folding classes (α, β, and αβ) analysed. 15

**SI REFERENCES** 17

SUPPORTING INFORMATION TABLES

**SI Table 1.** ProteinLIPs’ browsers compatibility. Minimum versions of browsers tested where ProteinLIPs works properly.

| **OS** | **Version** | **Chrome** | **Firefox** | **Microsoft Edge^a^** | **Safari^a^** |
| --- | --- | --- | --- | --- | --- |
| Linux | Ubuntu 16.04 | 60 | 60 | n/a | n/a |
| MacOS | 10.11 | 60 | 60 | n/a | 10 |
| Windows | 10 | 60 | 60 | 12 | n/a |
| ^a^ n/a: not applicable. | | | | | |

| **SI Table 2.** The 50 protein domains from CATH (Knudsen and Wiuf, 2010) used for the analysis of LIPs composition and enrichment. | | | | | | | | | | | | | | | | | | | | | | | | | | | | | |
| --- | --- | --- | --- | --- | --- | --- | --- | --- | --- | --- | --- | --- | --- | --- | --- | --- | --- | --- | --- | --- | --- | --- | --- | --- | --- | --- | --- | --- | --- |
| **Folding Class^a^** | **Superfamily^b^** | **Domain 1** | | | | **Domain 2** | | | | **Domain 3** | | | | | | | **Domain 4** | | | | | | | | **Domain 5** | | | | |
|  |  | **PDB** | **Chain** | **AAs Range** (length) | **Organism** | **PDB** | **Chain** | **AAs Range** (length) | **Organism** | **PDB** | **Chain** | | | **AAs Range** (length) | **Organism** | | **PDB** | **Chain** | | **AAs Range**  (length) | | **Organism** | | | **PDB** | **Chain** | | **AAs Range**  (length) | **Organism** |
| α | Tyrosine-protein kinase receptor  (1.10.510.10) | 6NSS | A | 595-795 (201) | *H. sapiens* | 6I8Z | A | 506-688 (183) | *H. sapiens* | 6GQQ | | A | 923-1167 (245) | | *H. sapiens* | 5VO2 | | | A | | 197-398 (202) | | *H. sapiens* | 5YA5 | | | A | 1163-1345 (183) | *H. sapiens* |
|  | Winged helix DNA-binding domain (1.10.10.10) | 3KFW | X | 3-66 (64) | *M. tuberculosis* | 1XMK | A | 288-366 (79) | *H. sapiens* | 1IUY | | A | 1-91 (91) | | *Mus musculus* | 1AOY | | | A | | 1-78 (78) | | *E. coli K-12* | 4QLC | | | U | 22-97 (76) | *Gallus gallus* |
|  | Aspartate receptor, ligand-binding domain  (1.20.120.30) | 1U89 | A | 751-889 (139) | *Mus musculus* | 2L7A | A | 781-911 (131) | *Mus musculus* | 2B0H | | A | 1837-1973 (137) | | *Mus musculus* | 4P9T | | | D | | 144-260 (117) | | *Mus musculus* | 5H5M | | | B | 380-501 (122) | *C. elegans* |

| **SI Table 2.** Continuation… | | | | | | | | | | | | | | | | | | | | | |
| --- | --- | --- | --- | --- | --- | --- | --- | --- | --- | --- | --- | --- | --- | --- | --- | --- | --- | --- | --- | --- | --- |
| **Folding Class^a^** | **Superfamily^b^** | **Domain 1** | | | | **Domain 2** | | | | **Domain 3** | | | | **Domain 4** | | | | **Domain 5** | | | |
|  |  | **PDB** | **Chain** | **AAs Range**  (length) | **Organism** | **PDB** | **Chain** | **AAs Range**  (length) | **Organism** | **PDB** | **Chain** | **AAs Range**  (length) | **Organism** | **PDB** | **Chain** | **AAs Range**  (length) | **Organism** | **PDB** | **Chain** | **AAs Range**  (length) | **Organism** |
| β | Trypsin-like serine proteases  (2.40.10.10) | 5LYO | C | 743-855 (113) | *H. sapiens* | 6FID | A | 137-246 (110) | *Bos taurus* | 1A7S | A | 1-103 (103) | *H. sapiens* | 6QHC | B | 135-244 (110) | *H. sapiens* | 2XXL | B | 246-380 (135) | *D. melanogaster* |
|  | Immunoglobulins (2.60.40.10) | 1WGO | A | 27-123 (97) | *H. sapiens* | 1X3D | A | 17-110 (94) | *H. sapiens* | 4U7K | H | 685-770 (86) | *H. histolytica* | 1AXI | B | 128-236 (109) | *H. sapiens* | 2WV3 | A | 3-91 (89) | *R. norvegicus* |
|  | SH3 Domains (2.30.30.40) | 2DL8 | A | 1-72 (72) | *H. sapiens* | 2DL5 | A | 1-78 (78) | *H. sapiens* | 2KE9 | A | 282-348 (67) | *H. sapiens* | 2W10 | B | 1-62 (62) | *Mus musculus* | 2EQI | A | 1-69 (69) | *Mus musculus* |

| **SI Table 2.** Continuation… | | | | | | | | | | | | | | | | | | | | | |
| --- | --- | --- | --- | --- | --- | --- | --- | --- | --- | --- | --- | --- | --- | --- | --- | --- | --- | --- | --- | --- | --- |
| **Folding Class^a^** | **Superfamily^b^** | **Domain 1** | | | | **Domain 2** | | | | **Domain 3** | | | | **Domain 4** | | | | **Domain 5** | | | |
|  |  | **PDB** | **Chain** | **AAs Range**  (length) | **Organism** | **PDB** | **Chain** | **AAs Range**  (length) | **Organism** | **PDB** | **Chain** | **AAs Range**  (length) | **Organism** | **PDB** | **Chain** | **AAs Range**  (length) | **Organism** | **PDB** | **Chain** | **AAs Range**  (length) | **Organism** |
| αβ | Phosphorylase kinase; domain 1  (3.30.200.20) | 3ULZ | A | 272-573 (302) | *A. thaliana* | 4E93 | A | 553-822 (270) | *H. sapiens* | 6D8E | A | 696-1019 (324) | *H. sapiens* | 4GYI | A | 92-342 (251) | *C. thermophilum* | 1FMK | A | 262-340 (79) | *H. sapiens* |
|  | NAD(P)-binding Rossmann-like domain  (3.40.50.720) | 1VPD | A | 3-166 (164) | *S. enterica* | 2OFP | B | 1-168 (168) | *E. coli K-12* | 3F3S | B | 6-187 (182) | *H. sapiens* | 3K6J | A | 6-215 (210) | *C. elegans* | 5UQ9 | H | 2-176 (175) | *H. sapiens* |
|  | Glutamine Phosphoribosylpyrophosphate, subunit 1, domain 1 (3.60.20.10) | 6AVO | X | 11-213 (203) | *H. sapiens* | 5FMG | Z | 1-193 (193) | *P. falciparum* | 6QM8 | A | 33-249 (217) | *L. tarentolae* | 5JI3 | D | 1-174 (174) | *E. coli* | 3MKA | Z | 301-522 (222) | *M. tuberculosis* |

| **SI Table 2.** Continuation… | | | | | | | | | | | | | | | | | | | | | |
| --- | --- | --- | --- | --- | --- | --- | --- | --- | --- | --- | --- | --- | --- | --- | --- | --- | --- | --- | --- | --- | --- |
| **Folding Class^a^** | **Superfamily^b^** | **Domain 1** | | | | **Domain 2** | | | | **Domain 3** | | | | **Domain 4** | | | | **Domain 5** | | | |
|  |  | **PDB** | **Chain** | **AAs Range**  (length) | **Organism** | **PDB** | **Chain** | **AAs Range**  (length) | **Organism** | **PDB** | **Chain** | **AAs Range**  (length) | **Organism** | **PDB** | **Chain** | **AAs Range**  (length) | **Organism** | **PDB** | **Chain** | **AAs Range**  (length) | **Organism** |
| αβ | Aspartate Aminotransferase, domain 1 (3.90.1150.10) | 4CVQ | B | 51-293 (243) | *E. coli K-12* | 2X5D | D | 51-293 (243) | *P. aeruginosa PAO1* | 5VER | B | 80-332 (253) | *Mus musculus* | 5VYE | D | 18-255 (238) | *P. putida* | 4WB0 | B | 57-312 (256) | *L. mexicana* |
| ^a^ The three main folding classes in CATH (Mainly Alpha, Mainly Beta and Alpha Beta) from which the superfamilies indicated in the second column were selected.  ^b^ CATH superfamily names and codes (between parentheses) from which the domains indicated in the contiguous columns on the right were selected. | | | | | | | | | | | | | | | | | | | | | |

| **SI Table 3.** Data related to protein domains from the initial dataset predicted to lack LIPs.^a^ | | | | | |
| --- | --- | --- | --- | --- | --- |
| **CATH Folding Class^b^** | **CATH Superfamily^c^** | **PDB** | **Chain** | **AAs Range (length)** | **Organism** |
| β | Trypsin-like serine proteases  (2.40.10.10) | 9EST | A | 132-245 (114) | *Sus scrofa* |
|  |  | 6MV4 | H | 130-230 (101) | *Homo sapiens* |
|  | Immunoglobulins (2.60.40.10) | 1RHF | B | 99-182 (84) | *Homo sapiens* |
| ^a^ The listed domains do not contain LIPs and were replaced―for the composition and enrichment analysis―with new domains of the same folding class that contain LIPs. They were, however, included in the dataset for evolutionary conservation (Consurf (Ben Chorin *et al.*, 2020)) and stability (SWOTein (Hou *et al.*, 2021)) analyses.  ^b^ Main folding class in CATH database (Knudsen and Wiuf, 2010) from which the superfamilies indicated in the second column were selected.  ^c^ CATH superfamily names and codes (between parentheses) from which the PDB indicated in the contiguous column was selected. | | | | | |

| **SI Table 4.** Default parameters for Consurf (Ben Chorin *et al.*, 2020) calculations. | | |
| --- | --- | --- |
| **Parameter** | | **Value** |
| Upload precalculated data | | |
|  | MSA | No |
|  | Tree | No |
| Homolog search | | |
|  | Algorithm | HMMER |
|  | # of iterations | 1 |
|  | E-value cutoff | 0.0001 |
|  | Protein data base | UNIREF-90 |
| Homolog selection | | |
|  | Mode | Automatic |
| Sequence selection | | |
|  | # of sequences | 150 |
|  | Algorithm | Sample the list of homologues |
|  | Maximal %ID between sequences | 95 |
|  | Minimal %ID for homologs | 35 |
| Multiple Sequence Alignment | | |
|  | Alignment method | MAFFT-L-INS-i |
| Evolutionary conservation calculation | | |
|  | Method | Bayesian |
|  | Evolutionary substitution model | Best model (default) |

| **SI Table 5.** Binding sites annotated in the analysed domains. | | | |
| --- | --- | --- | --- |
| **PDB** | **Site ID^a^** | **Ligand** | **Link** |
| 4E93 | AC1 | GUI | <https://www.rcsb.org/ligand/GUI> |
| 1axi | AC1 | SO4 | <https://www.rcsb.org/ligand/SO4> |
| 1vpd | AC1 | CL | <https://www.rcsb.org/ligand/CL> |
| 1vpd | AC2 | TLA | <https://www.rcsb.org/ligand/TLA> |
| 1xmk | AC1 | CD | <https://www.rcsb.org/ligand/CD> |
| 1xmk | AC2 | CD | <https://www.rcsb.org/ligand/CD> |
| 1xmk | AC3 | CD | <https://www.rcsb.org/ligand/CD> |
| 1xmk | AC4 | CD | <https://www.rcsb.org/ligand/CD> |
| 1xmk | AC5 | CD | <https://www.rcsb.org/ligand/CD> |
| 2ofp | AC3 | NAP | <https://www.rcsb.org/ligand/NAP> |
| 2ofp | AC4 | PAF | <https://www.rcsb.org/ligand/PAF> |
| 2ofp | AC6 | DIO | <https://www.rcsb.org/ligand/DIO> |
| 2ofp | AC7 | DIO | <https://www.rcsb.org/ligand/DIO> |
| 2ofp | AC8 | DIO | <https://www.rcsb.org/ligand/DIO> |
| 2ofp | AC9 | DIO | <https://www.rcsb.org/ligand/DIO> |
| 2w10 | AC1 | PO4 | <https://www.rcsb.org/ligand/PO4> |
| 2w10 | AC2 | PO4 | <https://www.rcsb.org/ligand/PO4> |
| 2x5d | AC1 | PLP | <https://www.rcsb.org/ligand/PLP> |
| 2x5d | AC2 | PLP | <https://www.rcsb.org/ligand/PLP> |
| 2x5d | AC9 | SO4 | <https://www.rcsb.org/ligand/SO4> |
| 2x5d | BC1 | SO4 | <https://www.rcsb.org/ligand/SO4> |
| 3f3s | AC6 | GOL | <https://www.rcsb.org/ligand/GOL> |
| 3f3s | AC7 | NAD | <https://www.rcsb.org/ligand/NAD> |
| 3f3s | AC9 | SO4 | <https://www.rcsb.org/ligand/SO4> |
| 3f3s | BC1 | CL | <https://www.rcsb.org/ligand/CL> |
| 3f3s | BC2 | CL | <https://www.rcsb.org/ligand/CL> |
| 3f3s | BC3 | CL | <https://www.rcsb.org/ligand/CL> |
| 3f3s | BC5 | GOL | <https://www.rcsb.org/ligand/GOL> |
| 3k6j | AC1 | PO4 | <https://www.rcsb.org/ligand/PO4> |
| 3k6j | AC2 | PO4 | <https://www.rcsb.org/ligand/PO4> |
| 3k6j | AC3 | PO4 | <https://www.rcsb.org/ligand/PO4> |
| 3k6j | AC4 | PO4 | <https://www.rcsb.org/ligand/PO4> |
| 3k6j | AC5 | ZN | <https://www.rcsb.org/ligand/ZN> |
| 4cvq | AC1 | PLP | <https://www.rcsb.org/ligand/PLP> |
| **SI Table 5.** Continuation… | | | |
| **PDB** | **Site ID^a^** | **Ligand** | **Link** |
| 4cvq | AC2 | PLP | <https://www.rcsb.org/ligand/PLP> |
| 4cvq | AC4 | ACT | <https://www.rcsb.org/ligand/ACT> |
| 4cvq | AC5 | GOL | <https://www.rcsb.org/ligand/GOL> |
| 4gyi | AC1 | ADP | <https://www.rcsb.org/ligand/ADP> |
| 4gyi | AC2 | MG | <https://www.rcsb.org/ligand/MG> |
| 4gyi | AC3 | EDO | <https://www.rcsb.org/ligand/EDO> |
| 4gyi | AC4 | EDO | <https://www.rcsb.org/ligand/EDO> |
| 4P9T | AD4 | EDO | <https://www.rcsb.org/ligand/EDO> |
| 4P9T | AD6 | IOD | <https://www.rcsb.org/ligand/IOD> |
| 4P9T | AD8 | IOD | <https://www.rcsb.org/ligand/IOD> |
| 4P9T | AE2 | EDO | <https://www.rcsb.org/ligand/EDO> |
| 4P9T | AE3 | EDO | <https://www.rcsb.org/ligand/EDO> |
| 4P9T | AE4 | PEG | <https://www.rcsb.org/ligand/PEG> |
| 4P9T | AE6 | IOD | <https://www.rcsb.org/ligand/IOD> |
| 4P9T | AE7 | IOD | <https://www.rcsb.org/ligand/IOD> |
| 4P9T | AE8 | IOD | <https://www.rcsb.org/ligand/IOD> |
| 4P9T | AE9 | EDO | <https://www.rcsb.org/ligand/EDO> |
| 4P9T | AF1 | EDO | <https://www.rcsb.org/ligand/EDO> |
| 4u7k | AC8 | CA | <https://www.rcsb.org/ligand/CA> |
| 4wb0 | AC1 | CAC | <https://www.rcsb.org/ligand/CAC> |
| 4wb0 | AC2 | CAC | <https://www.rcsb.org/ligand/CAC> |
| 5lyo | AC5 | SO4 | <https://www.rcsb.org/ligand/SO4> |
| 5lyo | AD4 | SO4 | <https://www.rcsb.org/ligand/SO4> |
| 5lyo | AD7 | SO4 | <https://www.rcsb.org/ligand/SO4> |
| 5lyo | AD8 | SO4 | <https://www.rcsb.org/ligand/SO4> |
| 5lyo | AD9 | SO4 | <https://www.rcsb.org/ligand/SO4> |
| 5ver | AC1 | PLP | <https://www.rcsb.org/ligand/PLP> |
| 5ver | AC2 | EPE | <https://www.rcsb.org/ligand/EPE> |
| 5ver | AC3 | PGE | <https://www.rcsb.org/ligand/PGE> |
| 5ver | AC5 | GOL | <https://www.rcsb.org/ligand/GOL> |
| 5ver | AC9 | PMP | <https://www.rcsb.org/ligand/PMP> |
| 5ver | AD1 | EPE | <https://www.rcsb.org/ligand/EPE> |
| 5ver | AD3 | GOL | <https://www.rcsb.org/ligand/GOL> |
| 5ver | AD4 | GOL | <https://www.rcsb.org/ligand/GOL> |
| **SI Table 5.** Continuation… | | | |
| **PDB** | **Site ID^a^** | **Ligand** | **Link** |
| 5ver | AD5 | CA | <https://www.rcsb.org/ligand/CA> |
| 5vo2 | AC1 | 9FV | <https://www.rcsb.org/ligand/9FV> |
| 5vye | AC4 | GOL | <https://www.rcsb.org/ligand/GOL> |
| 5vye | AC5 | PLR | <https://www.rcsb.org/ligand/PLR> |
| 5vye | AC7 | PLR | <https://www.rcsb.org/ligand/PLR> |
| 5vye | AC8 | GOL | <https://www.rcsb.org/ligand/GOL> |
| 6avo | AC1 | BZ7 | <https://www.rcsb.org/ligand/BZ7> |
| 6fid | AC2 | BEN | <https://www.rcsb.org/ligand/BEN> |
| 6fid | AC3 | SO4 | <https://www.rcsb.org/ligand/SO4> |
| 6fid | AC4 | SO4 | <https://www.rcsb.org/ligand/SO4> |
| 6fid | AC5 | SO4 | <https://www.rcsb.org/ligand/SO4> |
| 6gqq | AC1 | F8B | <https://www.rcsb.org/ligand/F8B> |
| 6i8z | AC1 | H82 | <https://www.rcsb.org/ligand/H82> |
| 6NSS | AC1 | L0M | <https://www.rcsb.org/ligand/L0M> |
| 6qhc | AC3 | DMS | <https://www.rcsb.org/ligand/DMS> |
| 6qhc | AC4 | 135 | <https://www.rcsb.org/ligand/135> |
| ^a^ Binding site nomenclature retrieved from the PDB files. | | | |
|  | | | |

| **SI Table 6.** Point-biserial correlation analysis for LIPs and secondary structure elements from Consurf (Ben Chorin *et al.*, 2020) (evolutionary conservation) and SWOTein (Hou *et al.*, 2021) (stability) per-residue scores.^a^ | | | |
| --- | --- | --- | --- |
| **Structure element** |  | **Consurf Score^b^** | **SWOTein Score (All)^c^** |
| mLIPs^d^ |  | 0.06* | 0.069* |
| cLIPs^d^ |  | ‒0.086* | ‒0.022 |
| Full LIPs^e^ |  | ‒0.029 | 0.026 |
| Alpha^f^ |  | 0.018 | ‒0.144* |
| Beta^f^ |  | ‒0.118* | 0.017 |
| Coil^f^ |  | 0.006 | 0.128* |
| ^a^ Correlation calculated between the conservation (Consurf) or stability (SWOTein) score and the binary classification of residues as either being inside (1) or outside (0) a given structure element (secondary structure or LIP).  ^b^ Positive correlations indicate a higher sequence conservation of residues in the indicated structure element compared to the rest of the protein. Negative correlations indicate a lower sequence conservation  ^c^ Original stability-related function values provided by SWOTein are positive for destabilizing contributions to global stability and negative for stabilizing contributions. To maintain this convention here, positive correlations indicate a destabilizing effect of the structure element, whereas negative correlations indicate a stabilizing effect. The ‘All’ term stands for the sum of SWOTein prediction values for ‘Distance’, ‘Accessibility’ and ‘Torsion’ stability components.  ^d^ mLIPs and cLIPs residues, as calculated by the ProteinLIPs server.  ^e^ Full LIPs encompass residues in mLIPs or cLIPs.  ^f^ Per-residue assignments of secondary structure as calculated by DSSP (Kabsch and Sander, 1983).  * $p_{value}<0.005$ using two tailed Student’s t-test for mean differences without multiple test correction. | | | |

| **SI Table 7.** Effect size analysis (Cohen’s *d*) for LIPs and secondary structure elements in the selected protein set from Consurf (Ben Chorin *et al.*, 2020) (evolutionary conservation) and SWOTein (Hou *et al.*, 2021) (stability) per-residue scores.^a^ | | | | |
| --- | --- | --- | --- | --- |
| **Structural element** |  | **Consurf Score** | **SWOTein Score (All)^b^** |  |
| mLIPs^c^ |  | 0.140 | 0.16 | |
| cLIPs^c^ |  | 0.183 | 0.046 | |
| Full LIPs^d^ |  | 0.058 | 0.053 | |
| Alpha^e^ |  | 0.039 | **0.303** | |
| Beta^e^ |  | **0.274** | 0.040 | |
| Coil^e^ |  | 0.013 | **0.280** | |
| ^a^ Results (*d* values) are formatted according to Cohen’s definition: small effect sizes: $0.2\leq d<0.5$ (highlighted in bold); medium effect sizes: $0.5\leq d<0.8$. No effect size is considered when $d\leq0.2$, while large effect size is recognised when $d\geq0.8$.  ^b^ The ‘All’ term stands for the sum of SWOTein prediction values for ‘Distance’, ‘Accessibility’ and ‘Torsion’ stability components.  ^c^ mLIPs and cLIPs residues as calculated by the *ProteinLIPs* server.  ^d^ Full LIPs encompass residues in mLIPs or cLIPs  ^e^ Per-residue assignments of secondary structure as calculated by the DSSP program (Kabsch and Sander, 1983). | | | | |

**SUPPORTING INFORMATION FIGURES**


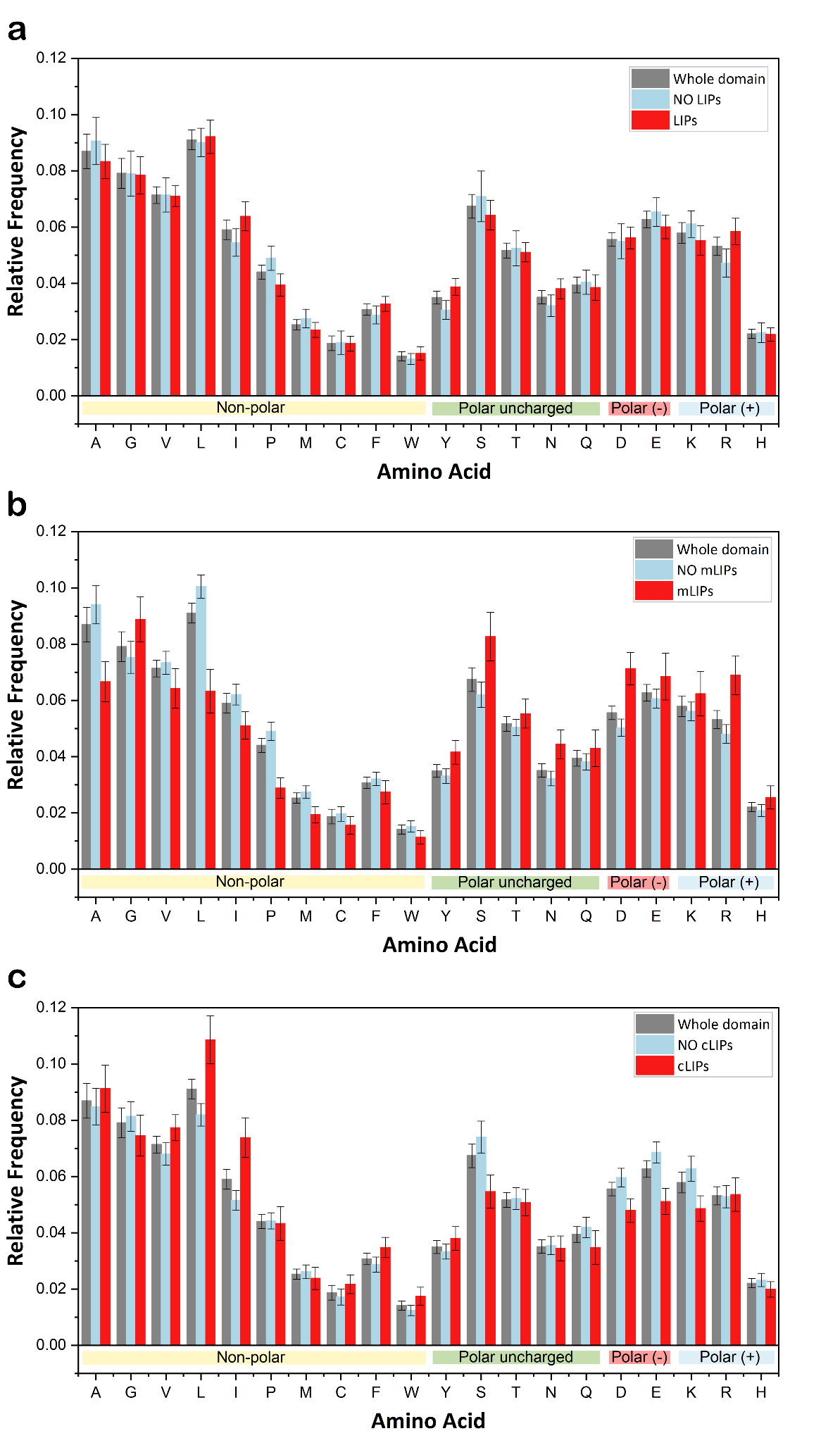


**SI Figure 1. Per amino acid frequency in LIPs across all the CATH folding classes (α, β, and αβ) analysed.** a) Frequencies obtained across entire LIPs (i.e., mLIPs+cLIPs). b) Frequencies obtained across mLIPs. c) Frequencies obtained across cLIPs. Error bars (standard errors) are represented on each residue bar. Coloured horizontal bars below the *x*-axis display the polarity group to which each amino acid belongs: pale yellow for non-polar, green for polar uncharged, red for positively charged, and pale blue for negatively charged.

**SI REFERENCES**

Ben Chorin,A. *et al.* (2020) ConSurf‐DB: An accessible repository for the evolutionary conservation patterns of the majority of PDB proteins. *Protein Sci*, **29**, 258–267.

Hou,Q. *et al.* (2021) SWOTein: a structure-based approach to predict stability Strengths and Weaknesses of prOTEINs. *Bioinformatics*, **37**, 1963–1971.

Kabsch,W. and Sander,C. (1983) Dictionary of protein secondary structure: Pattern recognition of hydrogen-bonded and geometrical features. *Biopolymers*, **22**, 2577–2637.

Knudsen,M. and Wiuf,C. (2010) The CATH database. *Hum Genomics*, **4**, 207–212.
